# Moiré Superlattice Effects and Band Structure Evolution in Near-30-Degree Twisted Bilayer Graphene

**DOI:** 10.1021/acsnano.1c06439

**Published:** 2022-01-24

**Authors:** Matthew J. Hamer, Alessio Giampietri, Viktor Kandyba, Francesca Genuzio, Tevfik O. Menteş, Andrea Locatelli, Roman V. Gorbachev, Alexei Barinov, Marcin Mucha-Kruczyński

**Affiliations:** † Department of Physics, University of Manchester, Oxford Road, Manchester M13 9PL, United Kingdom; ‡ National Graphene Institute, University of Manchester, Booth Street East, Manchester M13 9PL, United Kingdom; ¶ Elettra-Sincrotrone Trieste ScPA, Trieste 34149, Italy; § Henry Royce Institute, Oxford Road, Manchester M13 9PL, United Kingdom; ∥ Department of Physics, University of Bath, Claverton Down, Bath BA2 7AY, United Kingdom; ⊥ Centre for Nanoscience and Nanotechnology, University of Bath, Claverton Down, Bath BA2 7AY, United Kingdom

**Keywords:** twisted bilayer graphene, moiré superlattices, minigaps, photoemission, van Hove singularities, stacking-dependent electronic properties

## Abstract

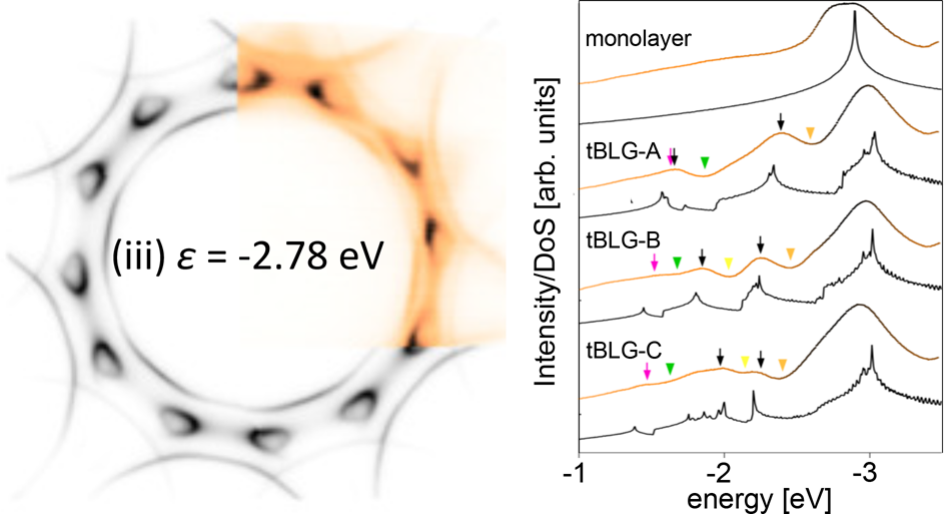

In stacks of two-dimensional crystals, mismatch of their lattice constants and misalignment of crystallographic axes lead to formation of moiré patterns. We show that moiré superlattice effects persist in twisted bilayer graphene (tBLG) with large twists and short moiré periods. Using angle-resolved photoemission, we observe dramatic changes in valence band topology across large regions of the Brillouin zone, including the vicinity of the saddle point at ***M*** and across 3 eV from the Dirac points. In this energy range, we resolve several moiré minibands and detect signatures of secondary Dirac points in the reconstructed dispersions. For twists θ > 21.8°, the low-energy minigaps are not due to cone anticrossing as is the case at smaller twist angles but rather due to moiré scattering of electrons in one graphene layer on the potential of the other which generates intervalley coupling. Our work demonstrates the robustness of the mechanisms which enable engineering of electronic dispersions of stacks of two-dimensional crystals by tuning the interface twist angles. It also shows that large-angle tBLG hosts electronic minigaps and van Hove singularities of different origin which, given recent progress in extreme doping of graphene, could be explored experimentally.

Twisted bilayer graphene (tBLG) is the archetype of van der Waals heterostructures—stacks of atomically thin materials with no directional bonding between consecutive layers and hence complete freedom of their relative rotational arrangement.^1,2^ Tuning the twist angle, θ, between lattice directions of neighboring crystals leads to formation of moiré superlattices (mSLs), represented visually by patterns observed, for example, with scanning probe techniques,^3−5^ and spatial modulation of interlayer coupling. This enables engineering of properties of a stack by tuning its stacking geometry, with examples including the observation of Hofstadter’s butterfly^6,7^ and interfacial polarons^8^ in graphene/hexagonal boron nitride heterostructures, as well as interlayer excitons in transition metal dichalcogenide bilayers.^9,10^ In tBLG, at small angles, θ ≈ 1°, mSLs generate flat bands which host correlated electronic behavior including superconductivity.^11,12^ At the maximum twist angle, θ = 30°, because the height-to-width ratio of a regular hexagon involves the irrational , tBLG is a quasicrystal.^13,14^ However, properties of tBLG with twist angles between these two limits remain relatively unexplored experimentally, with the current studies mainly focused on the van Hove singularity due to hybridization of Dirac cone crossings^15−19^ which can be tuned with electric fields^20,21^ and influences the optical properties of the stack.^20,22,23^

Here, we use angle-resolved photoemission spectroscopy (ARPES) to study evolution of the valence band structure of tBLG with large twist angles, θ ≳ 22°. We observe extensive modifications of the band structure not only near the intersections of the bands of the individual layers, but across a wide range of energies, ∼3 eV, away from the Dirac points: appearance of multiple minigaps and signatures of additional Dirac points appearing in the dispersion and hybridization of the isotropic bottoms of the graphene π-bands. We explain how these changes arise due to the coupling between the layers and mSL effects which persist at large twists when the apparent moiré wavelength is comparable to, but yet incommensurate with, the graphene lattice constant, and hence results in intervalley coupling. Our results demonstrate how, in a stack of two-dimensional crystals, the twist angle at an interface between two layers can be used to modify the electronic dispersion of the structure through a variety of mechanisms across a large range of θ. Moreover, given the successful extreme doping of monolayer graphene close to and past its ***M*** van Hove singularity,^24−27^ the richness of the band structure we observe suggests large-twist tBLG as a playground to explore interplay of interaction effects driven by van Hove singularities.

## Results and Discussion

We fabricated three tBLG devices, A, B, and C, on top of hexagonal boron nitride (*h*-BN) using exfoliation and dry peel stamp transfer technique.^28^ The tBLG samples were characterized by low-energy electron microscopy (LEEM) and low-energy electron diffraction (LEED) in order to determine the twist angles, θ = 22.6° (tBLG-A), 26.5° (tBLG-B), and 29.7° (tBLG-C). For large twist angles, using reciprocal space LEED patterns to measure the twist is more precise than investigating the real space moiré periodicity with the scanning probe techniques (widely used for small θ) because unit vectors for the latter are small, i.e., comparable with the graphene lattice constant. Our procedure, described in detail in the Supporting Information (SI), allows us to determine θ with the accuracy of 0.1°. In turn, by comparing the widths of the zeroth and first order LEED spots, we estimate the maximum twist angle disorder as Δθ = 0.2°. This indicates relative homogeneity of the twist angle across areas of our devices much larger than the nano-ARPES spot size (≲ 1 μm in diameter). The bottom graphene layer is rotated by θ_*h*–BN_ ≈ 10°, 15°, and 4° for the A, B, and C devices, respectively, with respect to the underlying *h*-BN—this is sufficient to avoid moiré effects at the *h*-BN/graphene interface which are the strongest in highly aligned *h*-BN/graphene structures^6,7^ and decrease with increasing θ_*h*-BN_.^29^ All the measurements were performed at the Elettra Synchrotron, and details of the fabrication process and discussion of the LEEM, LEED, and ARPES experiments are provided in the SI.

**Figure 1:**
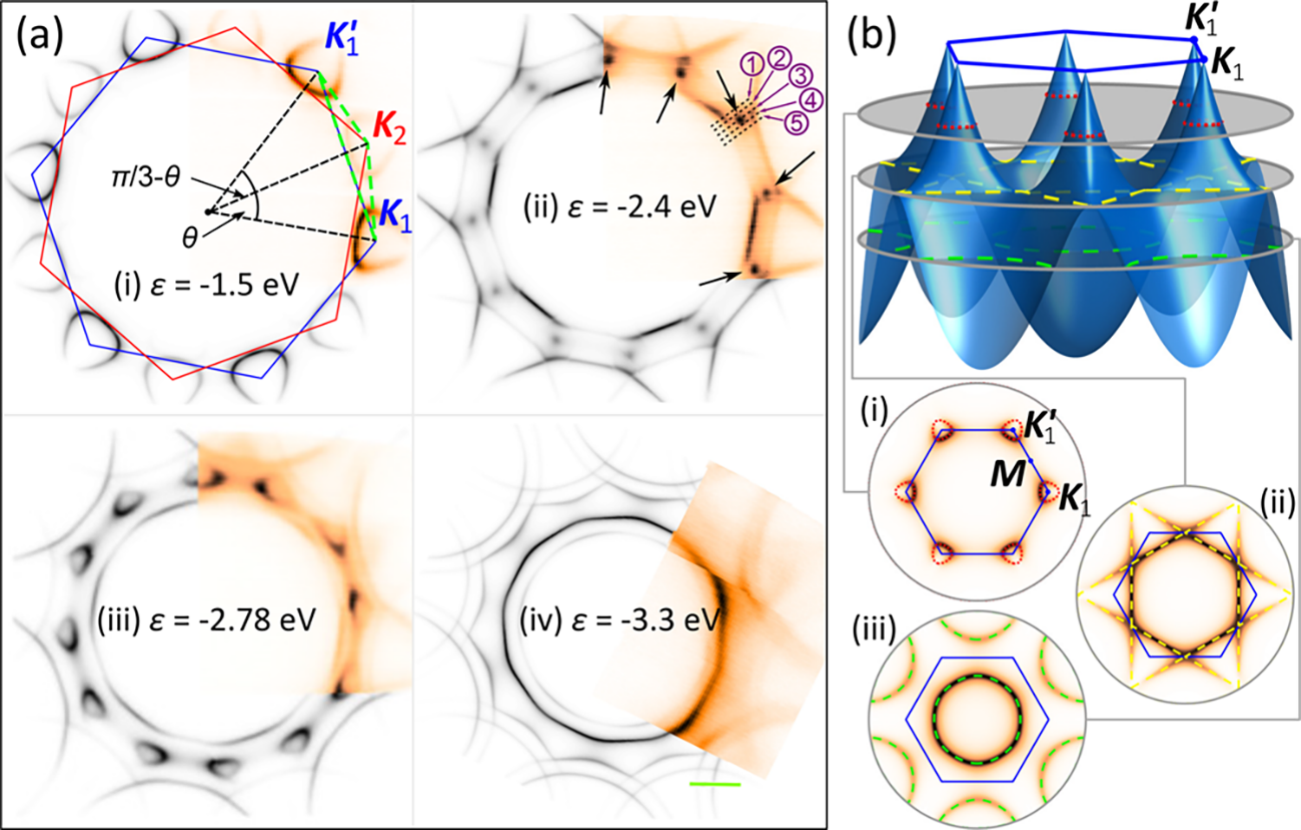
Topology of tBLG energy contours. (a) ARPES constant-energy maps of tBLG-C, θ = 29.7°; experimental data is shown in color and theoretical simulation in black and white. The blue and red hexagons show Brillouin zones of the top (*i* = 1) and bottom (*i* = 2) layers, respectively, and the green dashed line indicates the *k*-space path for cuts in Figure 3b. ***K***_*i*_ and ***K***_*i*_^′^ denote inequivalent Brillouin zone corners in layer *i*. Black arrows in panel ii point to secondary Dirac points; dashed black line segments numbered with purple numbers show paths of cuts presented in Figure 2. All panels show the same *k*-space area; the green scale bar in iv corresponds to 0.5 Å^–1^. (b, top) Valence band of MLG and its characteristic cross sections. The red dotted and yellow and green dashed lines show energy contours for cuts indicated by gray planes. (bottom) Simulated MLG ARPES constant-energy maps at energies of the cuts above. The saddle points in MLG dispersion are located at ***M***.

The importance of interlayer coupling and mSL effects in our structures is most strikingly captured by the constant-energy maps of ARPES intensity at energies ∼2.5 eV below the Dirac points of the layers, shown in Figure 1a for tBLG-C (experimental data in colors, simulation in black and white; we present constant-energy maps for tBLG-A and tBLG-B in SI). For comparison, evolution of the constant-energy line of monolayer graphene (MLG) is shown in Figure 1b. In undoped MLG, the constant-energy surface at the Fermi energy, ϵ = 0, consists of points, known as Dirac points, located at the corners of the hexagonal Brillouin zone (BZ) and marked as ***K***_1_ and ***K***_1_^′^ in the figure. For decreasing energy of the cut, each of the Dirac points gives rise to a closed contour, indicated with red dotted lines in the first plane cutting through the MLG dispersion in Figure 1b. Overall, two closed contours can be built from the pieces within the BZ, as seen in inset i below the MLG dispersion in which the contours and BZ shown in blue solid line are overlaid on the simulated ARPES intensity map for the same energy (note the crescent-like patterns of intensity around each valley, reflecting the topological nature of the Dirac points^30^). The contours grow away from the Dirac points and connect at the ***M*** points at the energy ϵ = ϵ_***M***_ corresponding to the position of cut ii in Figure 1b. For energies ϵ < ϵ_***M***_, cut iii, only one closed contour is present inside the BZ.

It is clear from the ARPES spectra in Figure 1a that the topology of large-angle tBLG bands is different. For energies 0 > ϵ ≳ – 1.5 eV, panel i, ARPES maps show 12 crescent-like shapes indicating twice the number of Dirac points, in agreement with the presence of two graphene layers. The six less intense features come from the bottom graphene layer, signal from which is attenuated due to the electron escape depth effect. At the energy ϵ ≈ −2.0 eV, the crescent shapes connect with each other and states belonging to different layers hybridize. This leads to the formation of one contour encircling the Γ point, similarly to MLG at ϵ < ϵ_***M***_, as well as, at energy ϵ ≈ −2.4 eV, panel ii, to additional intense features indicated with black arrows. These intense features evolve into new crescent shapes as shown in panel iii, ϵ = −2.78 eV, and the intensity patterns look strikingly similar to those in panels ai and bi, suggesting the presence of secondary Dirac points akin to those detected in small-angle tBLG^31^ or graphene aligned to underlying *h*-BN.^32−34^ The crescent-like patterns merge together at ϵ ≈ −3.1 eV so that for ϵ ≲ ϵ_***M***_, panel iv, the constant-energy maps contain two concentric contours. These are a consequence of hybridization of the approximately circular and degenerate bottoms of the π-bands of the two layers due to interlayer coupling with the states shifted to higher (lower) energies giving rise to the inside (outside) contour.

**Figure 2:**
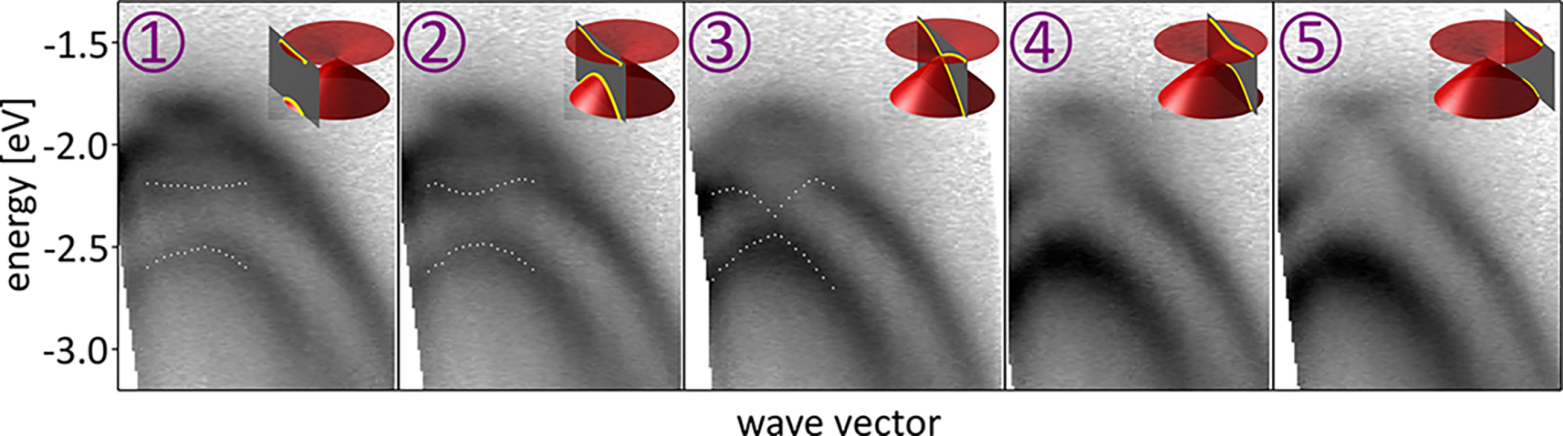
Secondary Dirac point in large-angle tBLG. Photoemission intensity along wave vector cuts in the vicinity of one of the secondary Dirac points discussed in the main text, as shown with black dashed lines and numbered in Figure 1aii. (insets) Schematically the shape of the two bands at the energy ∼−2.5 eV, with the gray planes indicating the location of the cut with respect to the sDP and the yellow lines highlighing the band cross-section for a given cut. The white dots in cuts 1–3 mark positions of Gaussian peaks fitted to the data to establish the band dispersion.

We investigate the secondary Dirac points from Figure 1aii in more detail by studying cuts marked 1–5 in that panel and show their photoemission maps in Figure 2. For cuts 1–3, we fitted the positions of two bands around the energy ∼−2.5 eV with Gaussians (see the SI for a description of the procedure), with their peaks as a function of wave vector marked with white dots. Our cuts suggest band structure feature containing a Dirac point as shown in the insets of each panel, where the gray planes indicate the location of the cut and the yellow lines highlight the band cut giving rise to the corresponding ARPES intensity. Our photoemission data cannot exclude the possibility that the secondary Dirac point is gapped; if so, the gap is smaller than ∼0.2 eV (limit imposed by our energy resolution and precision of the fitting procedure). Finally, while the symmetry of the constant-energy maps in Figure 1 implies that the band structure in cuts 1 and 2 is the same as in cuts 4 and 5, we do not see the band above the secondary Dirac point in the latter—this is because intensity from this part of the dispersion is affected by the Berry phase interference effects^30^ responsible for crescent-like intensity patterns from otherwise circular contours in the vicinity of Dirac points in the maps in Figure 1. In the SI, we show additional cuts in the vicinity of the new Dirac point in the direction roughly perpendicular to cuts in Figure 2.

**Figure 3:**
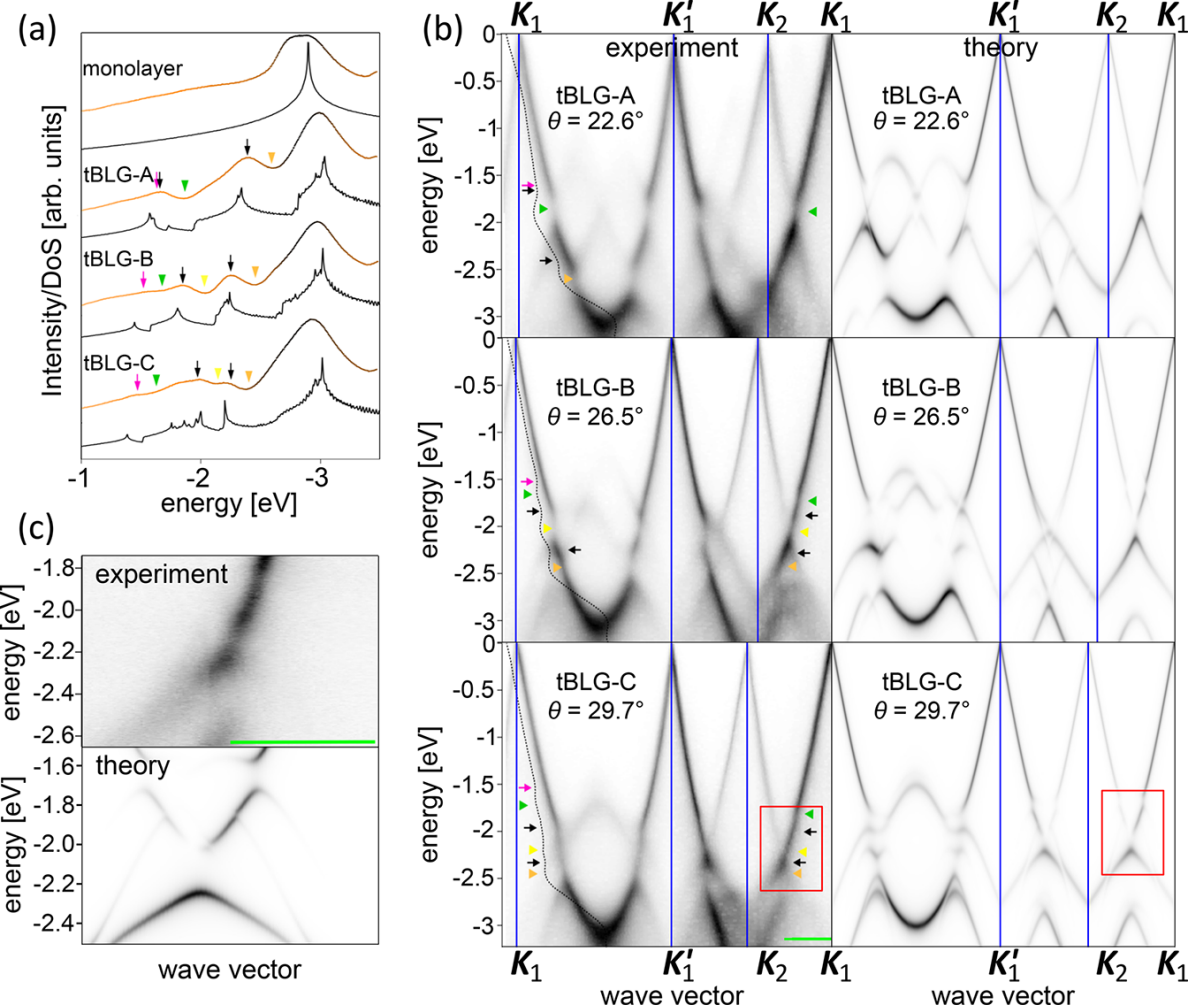
Minigaps in large-angle tBLG. (a) Energy distribution curves and simulated DoS for MLG and tBLG. Arrows and triangles indicate positions of vHs and minigaps with colors differentiating between the origin of the features as discussed in the text. (b) ARPES intensity along *k*-space path shown with green dashed line in Figure 1a, together with the corresponding theoretical simulation (right). The dotted lines are energy distribution curves from a with colored markers indicating the same features. (c) Closeup of the area marked with the red rectangle in b. The green scale bars in b and c correspond to 0.5 Å^–1^.

Changes in the topology of the constant-energy contours like these presented in Figure 1a are reflected by discontinuities in the electronic density of states (DoS): merging of two contours involves a saddle point and generates a van Hove singularity peak (vHs) while appearance of a new one generates a step due to a contribution from a new band. With this in mind, we study the photoemission energy distribution curves obtained by integration of the photocurrent across *k*-space. In Figure 3a, we compare the results for all tBLG samples as well as a reference monolayer region of one of the samples and DoS calculated using the continuum model^35−37^ (see the SI for a description of the theoretical model). The MLG DoS displays a single peak, in the ARPES data reflected as a broad “bump”, which corresponds to the saddle points at ***M***. The large width of this feature for MLG as compared to the theoretical DoS is due to the contribution from the valence band of *h*-BN with its band edge ∼2.7 eV below the graphene Dirac points responsible for the left side of the peak (while the *h*-BN signal is strongly attenuated for tBLG, this is less so for the MLG with only one graphene layer on top of the substrate). A similar feature originating from the ***M*** saddle points is also present at slightly shifted positions in all the tBLG DoS. However, the tBLG curves contain additional features indicating the presence of several vHs singularities and suggesting a more complicated band structure evolution than evident from the constant-energy maps. Positions of these features are well correlated with sharp peaks in the theoretical DoS below each experimental plot—we highlight with arrows the maxima and with triangles the minima of photocurrent that are of special interest below.

The top curve in Figure 3a was obtained by moving the nano-ARPES spot off the region where the two layers overlap. This provides a direct comparison between monolayer and twisted bilayer and suggests that the changes in the photocurrent measured from tBLG areas are purely due to the interlayer interaction. In van der Waals heterostructures with twisted interfaces, two mechanisms are known to induce DoS peaks: (i) direct hybridization of states from different layers^15^ and (ii) coupling between states backfolded by the mSL.^38^ Both lead to opening of gaps in the electronic spectrum as a consequence of coupling between electronic states, accompanied by the appearance of saddle points in the dispersion which in turn are responsible for the DoS peaks. Therefore, to understand the energy distribution curves in Figure 3a, we look for signs of minigap formation by investigating photoemission spectra along the *k*-space paths connecting the valleys ***K***_1_, ***K***_1_^′^, and ***K***_2_ as shown in Figure 1a. We present these cuts in Figure 3b, together with simulations produced using a model established for ARPES studies of graphene on *h*-BN^34^ and applied to graphene stacks^39,40^ (see the SI for details). The theoretical model captures all the qualitative features of the experimental data. Moreover, DoS minima in panel a coincide with the positions of the minigaps in panel b. Because opening of minigaps in the electronic spectrum of two-dimensional materials must be accompanied by generation of saddle points, we identify the DoS maxima with a vHs in the vicinity of each minigap. For devices tBLG-C and tBLG-B, we can resolve at least three minigaps, as shown in more detail for the former in panel c which presents a separate measurement of the region indicated by the red rectangle in b. This implies observation of four minibands, a testament of the outstanding quality of our samples.

**Figure 4:**
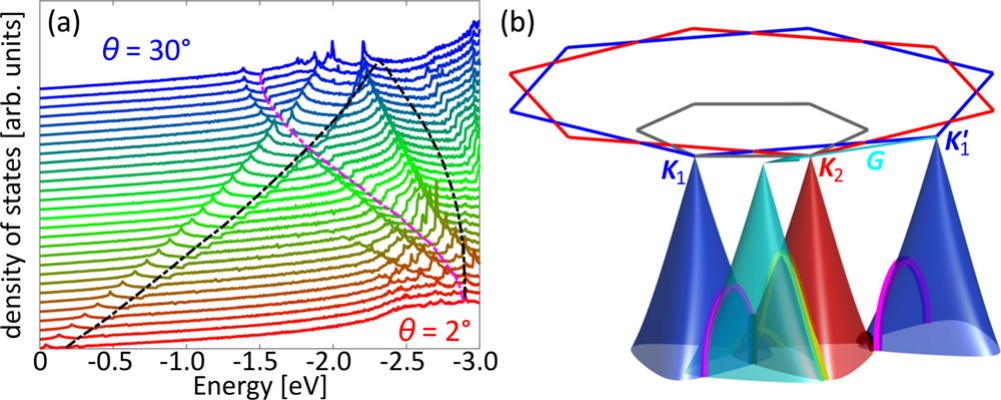
Moiré-induced scattering in large-angle tBLG. (a) Evolution of the tBLG DoS for θ = 2° (red) to θ = 30° (blue), in steps of 1° (curves shifted vertically for clarity). The dashed lines are guides for the eye indicating, for given θ, highest energies of the crossings marked with the corresponding color in b. (b) Hierarchy of crossings in tBLG with θ > θ_c_. The blue and red hexagons are the BZ of the top and bottom graphene layer; their valence band structures in the vicinity of ***K***_1_, ***K***_1_^′^, and ***K***_2_ are shown with blue and red surfaces, respectively. The cyan cone depicts the ***K***_1_^′^ states shifted by a moiré reciprocal vector ***G*** indicated with the cyan arrow (the moiré BZ is shown in gray). Crossings between MLG dispersions are highlighted in black (between two MLG dispersions twisted by θ), magenta (***K***_1_ cone and ***K***_1_^′^ translated by ***G***; ***K***_1_^′^ cone and ***K***_1_ translated by −***G***) and yellow (***K***_2_ cone of bottom MLG and ***K***_1_^′^ translated by ***G***).

To discover the origin of the observed minigaps and vHs, we study the evolution of DoS calculated for twists 2° ≤ θ ≤ 30°, in steps of 1°, shown in Figure 4a (curves have been shifted vertically for clarity). In the absence of interlayer coupling, two MLG dispersions rotated with respect to each other by θ must intersect and we mark such crossings in black in Figure 4b where we show conical valence band dispersions of the top (blue) and bottom (red) layers for θ = 26.5°. The neighboring Dirac points are separated by a distance (35) (***K***_1_ and ***K***_2_ as marked in Figure 1), where *a* is the graphene lattice constant, or  (***K***_2_ and ***K***_1_^′^). The highest energies of crossings occur midway between every pair of Dirac points and the corresponding energies as a function of θ are indicated with the black dashed lines on top of the DoS curves in Figure 4a. Interlayer coupling hybridizes the degenerate states at the crossings, turning them into anticrossings accompanied by a saddle point between the Dirac points and above the gap (note that the saddle point is shifted off the line connecting the Dirac points^38^) and a quasi-quadratic edge of the next miniband below. The corresponding DoS features, peak at higher energies due to the saddle point, and a step at lower energies due to the band edge, can be seen in the vicinity of both dashed black lines in the DoS curves in panel a (the hybridization minigap does not open a global band gap as other parts of the electronic dispersion overlap with it so that the electronic density of states does not go down to zero^15,17,36^). At small twist angles, the feature closest to the Dirac points is due to mixing of states between pairs of Dirac cones closest to each other and has been studied using scanning tunnelling spectroscopy,^15,17^ ARPES,^16,23^ and magnetic focusing.^31^ At larger twists, separations between all pairs of neighboring Dirac cones become comparable, driving the associated vHs into the energy range ∼2 eV from the Dirac points. Guided by the approximate positions of minigaps indicated by the black dashed lines in Figure 4a, we ascribe the ARPES features marked with black arrows in Figure 3a to vHs formed above direct-hybridization gaps while the gaps themselves correspond to features indicated with the yellow and orange triangles.

Interestingly, for tBLG-B and tBLG-C, the ARPES features marked in Figure 3a with magenta arrows and green triangles cannot be explained by mixing of degenerate electronic states of the two layers by interlayer coupling. Instead, they evidence scattering of electrons by the mSL. In Figure 4b, we show in gray the moiré BZ in relation to the BZ of the graphene layers (red and blue for bottom and top, respectively). The primitive reciprocal vectors of the mSL correspond to the shortest vectors produced by subtraction of the reciprocal vectors of the two crystals,^35,41^ with one such vector, ***G***, portrayed by the cyan arrow. Scattering of electrons from the valley ***K***_1_^′^ of the top layer by that moiré reciprocal vector can be schematically depicted by translating the whole cone, producing the cyan surface which intersects with conical dispersion surfaces of the top layer around ***K***_1_. We mark this intersection with a magenta line on the ***K***_1_ cone. We also mark in the same color on ***K***_1_^′^ cone the equivalent intersection of ***K***_1_^′^ dispersion with ***K***_1_ translated by −***G***. The highest energy of these crossings, midway between ***K***_1_ and translated ***K***_1_^′^ (or between ***K***_1_^′^ and translated ***K***_1_) is indicated as a function of θ with the dashed magenta line in Figure 4a and provides an estimate for the position of a vHs formed above a minigap opened due to the moiré-induced intervalley interaction of ***K***_1_^′^ electrons with those in ***K***_1_. For small twists, the primitive reciprocal vectors of mSL are short and the ***K***_1_^′^ replica intersects the original dispersion of the top layer far below the Dirac points. The energy of the intersection increases with increasing twist angle as the moiré reciprocal vector scatters ***K***_1_^′^ electrons closer to ***K***_1_. At , the distance between ***K***_1_ and ***K***_1_^′^ replica is the same as between ***K***_1_ and ***K***_2_ so that the highest energies of the corresponding intersections are at similar energies (the energies are not identical because of the trigonal warping of the cone-like dispersions). This means that the related minigaps and vHs should also overlap as is indeed the case for DoS of sample tBLG-A with θ = 22.6° in Figure 3a. For larger twist angles, scattering on the moiré potential brings the ***K***_1_^′^ states close enough to ***K***_1_ so that it is this process, rather than direct hybridization of ***K***_1_ and ***K***_2_ cones, that is responsible for the ARPES features closest to the Dirac points in tBLG-B and tBLG-C: minigaps indicated with green triangles and vHs marked with magenta arrows in Figure 3.

**Figure 5:**
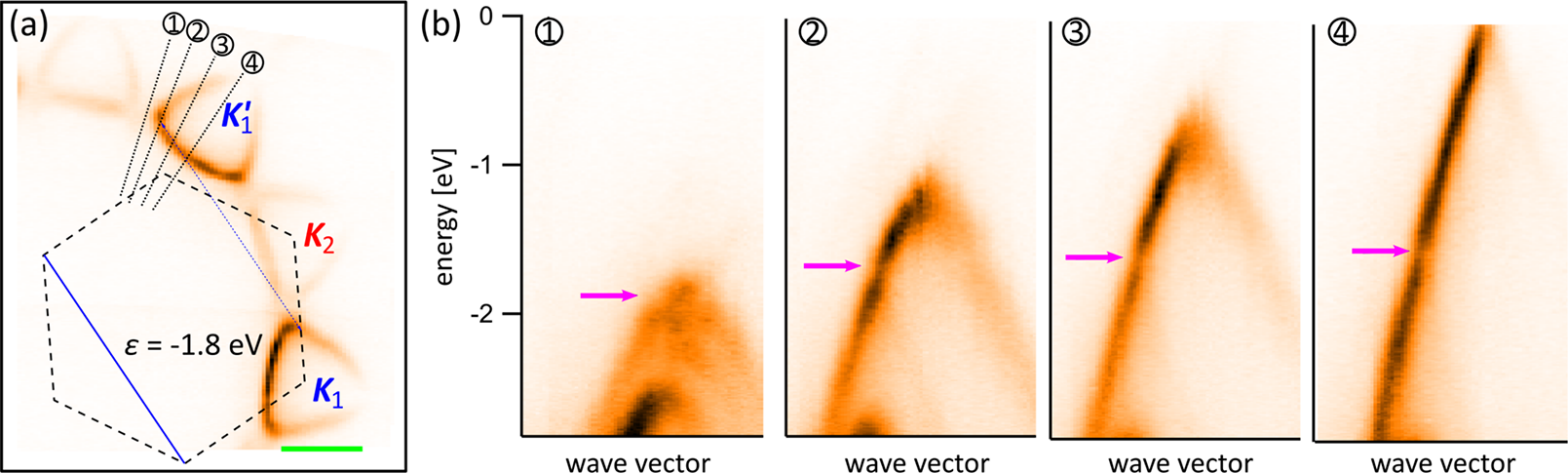
Tracking mSL minigaps in electronic dispersion. (a) Constant-energy map for tBLG-C for energy ϵ = −1.8 eV showing coupling between states related by a moiré reciprocal vector (thin blue line). The moiré BZ is drawn in black dashed lines with the same moiré reciprocal vector presented in blue for comparison. The green scale bar corresponds to 0.5 Å^–1^. (b) Photointensity is measured along cuts 1–4 marked in panel a. The magenta arrows indicate, for each cut, the position of the minigap formed due to the moiré-induced coupling between states in the ***K***_1_ and ***K***_1_^′^ valleys of the top graphene layer.

Further confirmation that moiré-induced scattering is responsible for some of the minigaps and vHs we observe can be provided by explicitly connecting affected states with mSL reciprocal vectors. In the constant-energy map in Figure 5a, corresponding to the energy marked by the green triangle for tBLG-C in Figure 3b, ϵ = −1.8 eV, we connect positions of the minigaps around ***K***_1_ and ***K***_1_^′^ with the moiré reciprocal vector ***G*** (thin blue line; see SI for procedure used to determine the moiré BZ). Moreover, in panel b we show photoemission measured along the cuts 1–4 as numbered and marked in a. Using these cuts, we can trace the crossing of ***K***_1_^′^ cone with the ***K***_1_ one translated by −***G*** and the resulting minigap, indicated with the magenta arrow for each cut, effectively following the magenta line on the ***K***_1_^′^ cone in Figure 4b. Note that we do not observe any minigaps (or features in the experimental and theoretical DoS) due to the hypothetical crossings between ***K***_1_^′^ states scattered by moiré superlattice and bottom layer dispersion around ***K***_2_ (yellow line in Figure 4b). This is because such a process is higher order in the mSL perturbation (it involves additional interlayer tunnelling). Finally, scattering of bottom layer electrons on the potential of the top layer (moiré-induced coupling between ***K***_2_ and ***K***_2_^′^; not shown in Figure 4b) is difficult to observe because of the additional attenuation of the signal from the bottom layer.

With regards to the magnitudes of the minigaps, the largest direct hybridization gap we observe is the one for tBLG-C shown in Figure 3c, Δ_direct_ ∼ 0.25 eV. For intermediate twist angles, 1° ≪ θ ≪ 30°, an estimate of this gap can be obtained via degenerate perturbation theory for two states coupled by *t* ≈ 0.11 eV,^35,36^ yielding Δ_direct_ = 2*t* ≈ 0.22 eV. At small angles, the moiré wave vector rapidly decreases with decreasing twist angle so that mSL couples states on Dirac cones with energy separation ϵ ≪ *t*. Level repulsion between these densely packed states leads to miniband separations decreasing with θ and flat bands in the extreme limit of the magic angle.^36^ In turn, at large angles, as discussed earlier the moiré vectors are sufficiently long to couple the two degenerate states to states from other valleys, some with energies within ∼*t* from the band crossing. Such states lead to a slight increase of the hybridization minigap with angle and a complicated band structure in its vicinity with several additional (moiré-induced) minigaps as observed for tBLG-B and tBLG-C (in the limit of θ = 30°, moiré couples 12 equi-energetic states from both graphene layers^42^). To estimate the size of the minigap opened due to moiré-induced scattering, Δ_moiré_, one must consider at least three states: two degenerate states on the crossing of the ***K***_1_ and ***K***_1_^′^ + ***G*** cones (magenta line at the crossing of the blue and cyan cones in Figure 4b) and an electronic state of the bottom layer at the same wave vector and at energy Δϵ ∼ 1 eV away. The first two states are not directly coupled to each other but only to the third one through the interlayer coupling *t*, so that  eV. Note that such a minimal three-level model underestimates the moiré-induced gaps we observe. We discuss our estimates for Δ_direct_ and Δ_moiré_ in more detail in the SI.

We have checked that the suppression of photocurrent we identify with spectral minigaps cannot be ascribed to photoemission final state effects^43^ which include dependence of the intensity on photon energy as well as polarization.^44−48^ In the SI, we show single-particle spectral weight of the electronic wave function for the wave vector and energy range as used for the ARPES spectra in Figure 3c. This spectral weight contains all of the minigaps discussed here which demonstrates that these are true spectral features and do not arise as a result of suppression of photointensity due to the Berry phase or final state effects. Moreover, we have performed measurements using photons both with energies 27 and 74 eV and observed little change in the spectra (a comparison of a cut along the ***K***_1_ – ***K***_1_^′^ direction for sample tBLG-C measured at both photon energies is shown in the SI). We use linearly polarized light and our geometry is such that when measuring along the **Γ** – ***K***_1_ direction the detector is in the plane of incidence and the incident light is *p*-polarized. This determines directions in the reciprocal space along which the photointensity is suppressed due to the Berry phase associated with the BZ corners^30,44−47^ (in the valence band, starting from a BZ corner in the direction away from Γ, as evident in the maps in Figure 1ai) and allows us to confirm that these do not overlap with locations of the minigaps, see for example Figure 5. Our observations also cannot be the result of secondary scattering of photoelectrons as this leads to band replicas but not gap opening.^34^

## Conclusions

Our results demonstrate the robustness of the moiré superlattice picture at large twist angles when the moiré wavelength  is comparable to the graphene lattice constant, *a*, and cannot correspond to a lattice constant of a commensurate superlattice. In large-angle twisted bilayer graphene with θ > 21.8°, gaps opened by the moiré, together with the associated van Hove singularities, are the closest to the Dirac points density of states features evidencing interaction of the two graphene layers. The direct hybridization minigap is located deeper in the valence band and is the largest for tBLG-C with the twist close to 30°, Δ_direct_ ∼ 0.25 eV. The interlayer coupling also modifies the topology of the dispersion at energies in the vicinity of and below the Brillouin zone ***M*** points: we observe secondary Dirac points in the reconstructed spectrum as well as hybridization of the bottom parts of the valence bands. It is worth noting that the LEED spectra shown in the SI indicate no strain reconstruction in our graphene crystals.

While a signature of moiré-induced scattering was observed previously for a 30° twisted bilayer graphene with its aperiodic moiré,^13^ we show that these processes are not restricted to this special twist angle but rather provide a robust way of coupling electronic states, with the twist angle controlling the affected regions of reciprocal space. The recent work on graphene on InSe^49^ suggests that moiré-induced scattering is not limited to twisted bilayer graphene.

Finally, it has been shown that it is possible to dope MLG sufficiently to move the chemical potential to the ***M*** point van Hove singularity,^24−27^ and so it might be feasible to explore large-angle tBLG in a similar regime. Interestingly, a superconducting instability was predicted for the MLG doped to the vHs^24^ but magnetic ordering for tBLG doped to the Dirac cone anticrossing^50^ (situation not equivalent to magic-angle tBLG in which states coupled by moiré reciprocal vectors contribute significantly to the flat bands^36^), with recent experimental studies in agreement with the latter.^51^ This suggests large-angle tBLG as a platform in which the interaction effects at vHs of different origin (in-plane nearest-neighbor coupling, interlayer Dirac cone anticrossing, moiré-induced intralayer intervalley coupling) and competition between them could be explored.

## Methods

First, laterally large (>100 μm) and thin (<100 nm) *h*-BN was mechanically exfoliated onto a Ti/Pt (2/10 nm) coated highly *n*-doped silicon wafer. Monolayer graphene was then transferred onto the *h*-BN using the poly(methyl methacrylate) (PMMA) dry peel stamp transfer technique.^28,52^ To note, few-layer graphene (connect to the monolayer) overlapped the edge of the *h*-BN to form a ground to the highly conductive Ti/Pt/Si substrate. A second graphene flake was then deterministically transferred onto the stack to create the tBLG. The stack was then annealed at 300 °C for 3 h to allow contamination trapped between flakes to agglomerate through the self-cleaning mechanism.^53^ The LEEM, LEED, and ARPES measurements were performed at the Elettra Synchrotron.^54,55^ All ARPES spectra in the main text were obtained using photons with energy of 74 eV, except Figure 3c which has been obtained with 27 eV photons.

To simulate the ARPES spectra, we used the tight-binding model to describe each of the graphene layers coupled with a continuum description of the interlayer interaction.^35−37^ The layers are considered rigid (which is a good approximation at large angles for which variation of the interlayer distance across the moiré unit cell decreases to ≃0.01 Å^56^) and the interlayer coupling is taken into account via the Fourier transform of the Slater-Koster-like^57^ hopping between *p*-orbitals. Values of the parameters in our model are based on those used previously in the literature and are applicable to a large range of twist angles^37,39^ as well as the fit to the experimental ARPES data. The detailed discussion of the procedure used to produce photocurrent intensity is presented in the SI.

## Supporting Information Available

Further details of the LEEM, LEED, and ARPES measurements, determination of the twist angle, constant-energy ARPES maps for tBLG-A and tBLG-B, additional ARPES spectra for cuts across the secondary Dirac point and comparison of spectra taken with photons of different energy; description of the theoretical model used to obtain tBLG bands and ARPES spectra; effective model used to estimate magnitudes of the observed minigaps (PDF)
