# Supplementary material for: Moiré Superlattice Effects and Band Structure Evolution in Near-30-Degree Twisted Bilayer Graphene
Source: ACS Nano. 2022 Jan 24;16(2):1954–62. doi: 10.1021/acsnano.1c06439 (PMC9007532; doi:10.1021/acsnano.1c06439)
Supplement: Supplementary file 1 — nn1c06439_si_001.pdf [file nn1c06439_si_001.pdf]

Supporting Information: Moiré Superlattice Effects and Band Structure Evolution in Near-30-Degree Twisted Bilayer Graphene

Matthew J. Hamer,[†,‡] Alessio Giampietri,[¶] Viktor Kandyba,[¶] Francesca Genuzio,[¶] Tevfik O. Menteş,[¶] Andrea Locatelli,[¶] Roman V. Gorbachev,[†,‡,§] Alexei Barinov,[*,¶] and Marcin Mucha-Kruczyński[*,‖,⊥]

†*Department of Physics, University of Manchester, Oxford Road, Manchester, M13 9PL, United Kingdom*

‡*National Graphene Institute, University of Manchester, Booth Street East, Manchester, M13 9PL, United Kingdom*

¶*Elettra-Sincrotrone Trieste ScPA, Trieste 34149, Italy*

§*Henry Royce Institute, Oxford Road, Manchester, M13 9PL, United Kingdom*

‖*Department of Physics, University of Bath, Claverton Down, Bath BA2 7AY, United Kingdom*

⊥*Centre for Nanoscience and Nanotechnology, University of Bath, Claverton Down, Bath BA2 7AY, United Kingdom*

E-mail: Alexey.Barinov@elettra.eu; M.Mucha-Kruczynski@bath.ac.uk

Contents

Experimental measurements **3**

LEEM and μ-LEED 4

μ-ARPES 4

Twist angle measurements and Dirac cone replicas in LEED and ARPES maps . . 5

Constant-energy ARPES maps for tBLG-A and tBLG-B 7

Secondary Dirac point in the miniband spectrum 8

Comparison of spectra taken with photons of different energy 10

Theoretical model of minibands and photoemission spectra **11**

Electronic spectrum of twisted bilayer graphene 11

Estimate of moiré scattering gap size 13

ARPES simulations 16

Matching of simulations to experimental data 19

References **20**

Experimental measurements

Before the measurements the samples were transferred in ultra-high vacuum and annealed for several hours at 400-450°C to remove surface contamination. The measurements were performed in ultra-high vacuum of less than 2×10^{-10} mbar with the sample at room temperature for LEEM-LEED and at 95 K for ARPES.

While annealing can in some cases reorient graphene on h-BN,[1] we have no reason to think this has affected our samples in between LEED and ARPES measurements. The first annealing process was performed during fabrication and adjustments of the relative positions of the flakes, if any, happened at that point. We have performed ARPES and LEED measurements several times on the same locations, with the samples annealed before every measurement and did not observe any changes in the measured twist angles or ARPES spectra.

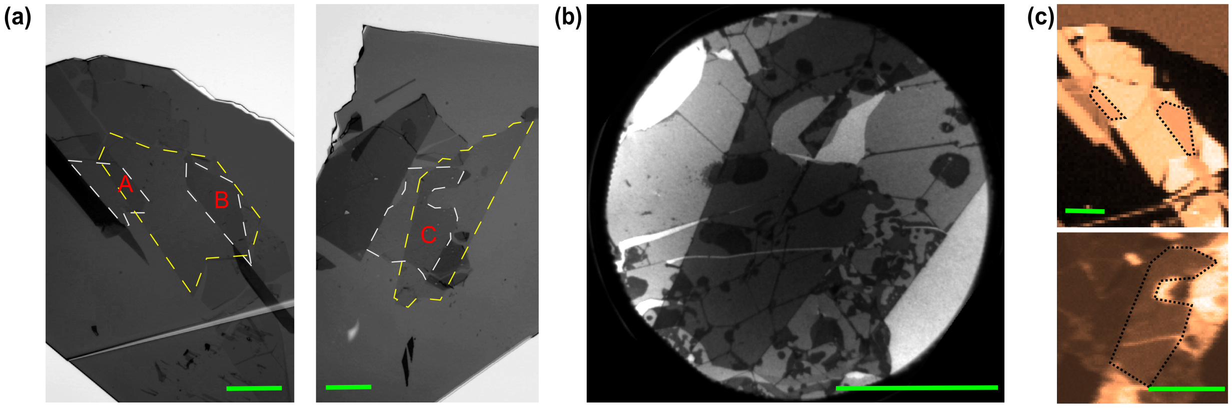

Figure S1: Selected optical (a), LEEM (b) and SPEM (c) images of the samples. In the optical micrographs, bottom graphene layers are delineated in yellow and the top graphene layers are in white. The overlap of the two layers determines tBLG-A, B and C devices as marked in the figure and delineated by black dotted polygons in (c). The scale bar in each image is 20 μm.

LEEM and μ-LEED

The sample morphology and crystal structure were studied using the SPELEEM microscope at the Nanospectroscopy beamline at Elettra.[2] In particular, low energy electron microscopy[3] was used to image the graphene and h-BN crystal grains, visualizing boundaries and defects [Fig. S1(b)]. In this manner, defect-free areas of homogeneous quality were selected for further LEED analysis. The LEED patterns were measured *in situ*, operating the microscope in diffraction mode. A suitable illumination aperture was used to limit the e-beam footprint on the sample, allowing us to probe a circular area of about 1 micron in diameter.

Careful analysis of the LEED patterns in Fig. S2 provides some information about the relative alignment between the h-BN and the bottom graphene layer. For example, for tBLG-C, a ring of six spots surrounding the brighter features of the bottom layer allows to obtain $\theta_{h-\mathrm{BN}}$.

μ-ARPES

In order to take angle resolved photoemission spectra from micrometer size areas on the samples (μ-ARPES), the synchrotron radiation light was focused to a 600 nm spot using Schwarzschild objectives with multilayer coated spherical mirrors optimized for photons of 27 and 74 eV. The photoelectron angle and energy distribution maps were obtained with a movable hemispherical electron energy analyzer.[4] To locate the region of interest for ARPES, scanning photoemission images (SPEM) were taken [Fig. S1(c)] with the angle and energy of the electron energy analyzer set to count selected graphene π-band(s): on the bottom image, the bottom layer graphene band distribution is shown; on top image the bands of both layers contribute to the intensity. Confronting the contrast variations in SPEM with optical and LEEM images allows to identify unambiguously the overlap of top and bottom graphene layers. The same points for both μ-LEED and μ-ARPES were selected to avoid possible differences in twist angle due to wrinkles and grains in exfoliated graphene flakes.

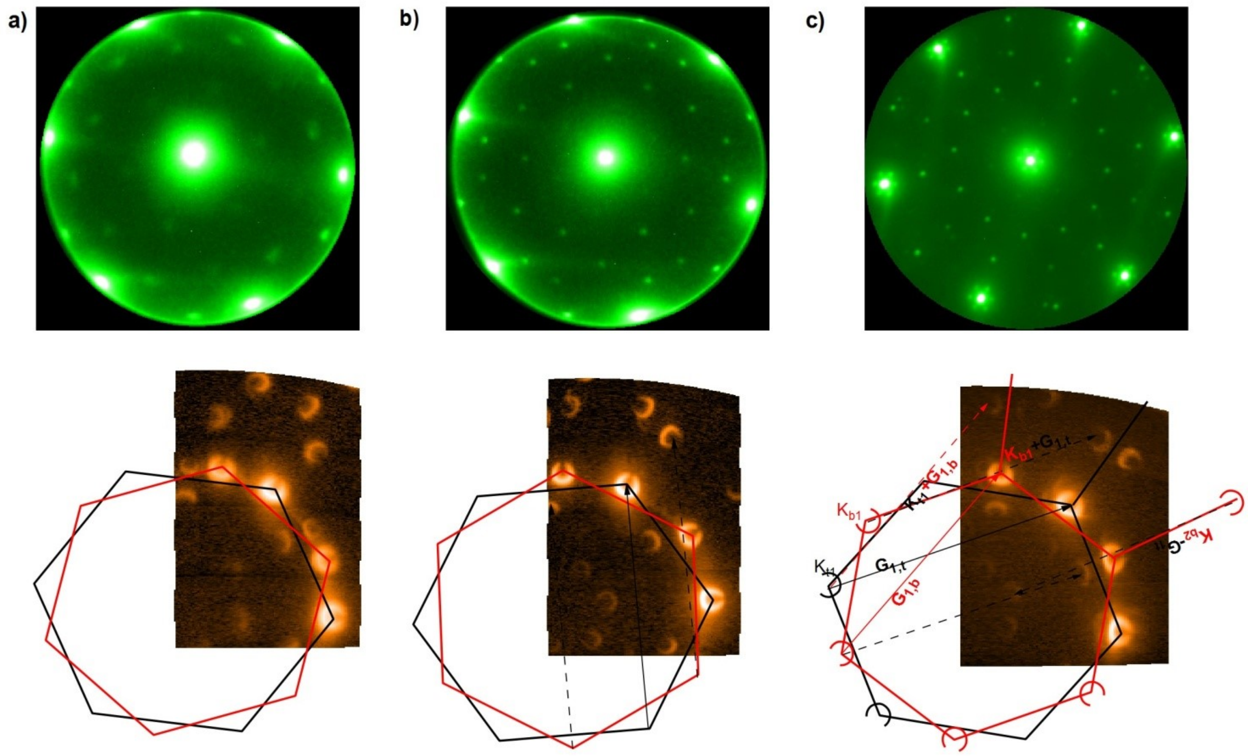

Figure S2: μ-LEED patterns with 45 eV electrons (top) and μ-ARPES constant energy maps at 0.8 eV (bottom) below DP for tBLG-A, B and C [panels (a), (b) and (c), respectively]. All intensities are plotted in logarithmic scale. BZs of top (bottom) layers are shown schematically as black (red) hexagons.

Twist angle measurements and Dirac cone replicas in LEED and ARPES maps

The twist angle θ can be selected during the fabrication process by aligning the edges of graphene flakes under the optical microscope. This leads to a potential error of 30°, depending on whether the edges of top and bottom layers are same or different of the two possible types, armchair or zigzag. Moreover, sub-degree resolution is necessary to compare theoretical calculations and experimental measurements reliably. For these reasons, μ-LEED patterns were used to determine θ for each sample. For large twist angles, this procedure, performed in the reciprocal space, is more precise than measuring the real space moiré periodicity with the scanning probe techniques (widely used for small θ) because real space moiré unit vectors are small, *i.e.* comparable with the graphene lattice constant.

The twist angles are calculated from LEED patterns shown in Fig. S2 by identifying *via* Gaussian fitting the coordinates of two hexagons formed by six main spots from each layer and then finding the rotation angle at which the sum of deviations of distances between pairs of reflections of different layers is minimal. The sum of clockwise and counter-clockwise twist angles found with this procedure was 60° within less than 0.1° for all three devices, which we therefore consider the accuracy of our twist angle measurement.

The maximum rotational disorder within the area of the μ-LEED spot can be evaluated by comparing the (Gaussian) width of the zeroth order diffraction spot (normal electron reflection not affected by rotational misalignment) to the width of 1st order peaks. We find the former to be 0.0166 $Å^{-1}$ while the latter is 0.0181 $Å^{-1}$ and 0.0173 $Å^{-1}$ for the top and bottom graphene layers, respectively. Assuming that the change in width is exclusively due to rotational disorder, its Gaussian distribution would have a width of 0.007 $Å^{-1}$ and 0.0049 $Å^{-1}$ for the top and bottom layers, respectively. This converted into to the angle gives $\Delta\theta_{\mathrm{top}} = 0.12°$ and $\Delta\theta_{\mathrm{bottom}} = 0.08°$, comparable with the accuracy of twist angle determination. Overall, the maximum twist disorder cannot be larger than $\Delta\theta = 0.2°$.

Slightly worse quality of the μ-LEED pattern from tBLG-A is likely because the region used for measurements was near exposed h-BN which was charging under the relatively large LEEM electron beam. In comparison, in the case of μ-ARPES, its sub-micron beam was not illuminating h-BN during the measurements.

All three devices show secondary LEED reflections and replicas of the characteristic crescent-like patterns in ARPES. In Fig. S2(c), the origin of several μ-ARPES replicas is illustrated: Dirac cones of the bottom layer (red) are scattered by the primitive reciprocal vectors of the top layer (black). The origin of the secondary reflections in LEED is similar. Such features have been initially observed for $\theta = 30°$.[5] Also visible in (c) are additional LEED reflections from the moiré pattern at the h-BN/graphene interface - out of all samples, for tBLG-C the underlying h-BN is aligned the closest with the bottom graphene layer ($\theta_{h-\mathrm{BN}} \approx 4°$).

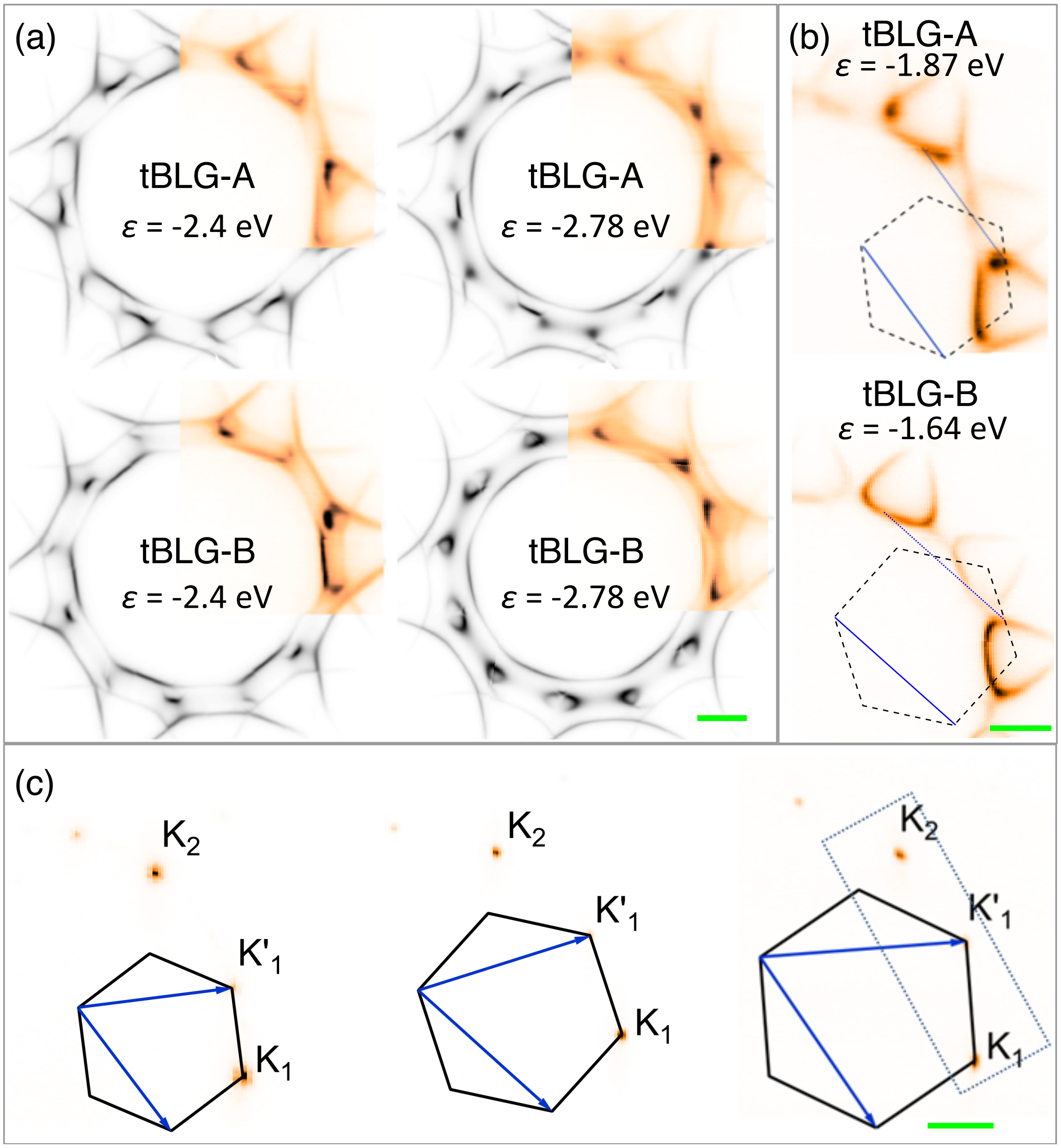

Figure S3: (a) Representative ARPES constant-energy maps for devices tBLG-A and B (coloured intensity represents measurement; black and white is simulated). (b) Examples of moiré-induced scattering between inequivalent valleys of the top graphene layer for tBLG-A and B. The black dashed hexagons denote the corresponding moiré Brillouin zone and the blue lines indicate one of the moiré primitive reciprocal vectors. (c) Constant-energy maps at $\epsilon = 0$ used to determine the size of the moiré Brillouin zones for all samples (from left to right, devices A to C). The dashed rectangle indicates the k-space area over which the ARPES signal was integrated to obtain the photointensity curves in Fig. 3(a) of the main text. The green scale bars in all panels correspond to 0.5 Å^{-1}.

Constant-energy ARPES maps for tBLG-A and tBLG-B

In Fig. S3(a) we present representative ARPES constant-energy maps for devices tBLG-A and tBLG-B which demonstrate that the evolution of their miniband spectra is qualitatively similar to that for tBLG-C presented in Fig. 1(a) of the main text. In particular, we observe similar crescent-like patterns at energies ~ 2.5 eV below the Dirac points suggesting the presence of secondary Dirac points in the electronic spectrum. In contrast to tBLG-C, these appear in two sets of six rather than one set of twelve, with each set forming at a different energy. This is because of the significant deviations of the twist angles from 30° and its 12-fold symmetry.

In panel (b), we show ARPES constant-energy maps with evidence of moiré-induced scattering in tBLG-A and tBLG-B. The black dashed hexagons denote the moiré Brillouin zones and the blue lines indicate the moiré primitive reciprocal vector which scatters electrons from one valley of the top layer to another (we plot this reciprocal vector twice to show how it fits within the moiré BZ and what electronic states it connects). The moiré-induced coupling leads to opening of minigaps which in the maps can be seen as interuption of the crescent-like intensity patterns.

Finally, in panel (c) we show constant-energy maps at $\epsilon = 0$ eV which were used to determine the $\boldsymbol{K}_1$-$\boldsymbol{K}_2$ distance and hence the effective moiré Brillouin zone for each tBLG sample. Once determined, this moiré BZ was then used for ARPES constant-energy maps at other energies, including the one shown in Fig. 5(a) of the main text. The dotted rectangle in the right-most figure shows k-space area which was integrated over to obtain the photointensity curves in Fig. 3(a) of the main text.

Secondary Dirac point in the miniband spectrum

In Fig. 2 of the main text, we show cuts through or in the vicinity of the secondary Dirac point we observe in the miniband spectrum of tBLG-C. For the cuts 1-3, we extract the dispersion of the two bands forming this Dirac point (white dots in the corresponding panels in the main text) by fitting the data with Gaussian peaks. We fitted all four bands visible

in the images. The widths of the four peaks were similar for the fit of panel 1 and 2 where all four bands have high intensity and can be identified separately. To fit the cut 3 where the intensity of the top band forming the secondary Dirac point considerably drops, we fixed the gaussian widths of the four bands to be as as in panels 1 and 2 to preserve consistency of the fit. Due to the lowered intensity of this band, the error in its position for this cut is ~ 0.2 eV, which we take as an error in the determination of the gap at the secondary Dirac point.

In Fig. S4, we present additional k-space cuts for tBLG-C in the vicinity of the secondary Dirac point discussed in the main text. These cuts, numbered 6 to 8, are roughly perpendicular to the cuts 1 to 5 shown in Fig. 2 of the main text. Directions of all the cuts are shown together for completeness in the first panel in Fig. S4. The intensity is shown in a linear scale from white to black and the spectra support the notion of a secondary Dirac point in the miniband structure at the energy $\epsilon_{\mathrm{sDP}} \approx -2.4$ eV below the original Dirac points at the graphene Brillouin zone corners. In the cut 7, which passes through one of the spectral features identified as the locations of the secondary Dirac points [see Fig. 1(a)(ii) of the main text], the two bands at the energy ϵ_{sDP} (red dashed arrow) come close to each other. For both the cuts 6 and 8, the distance betwen the bands is larger. Together with the

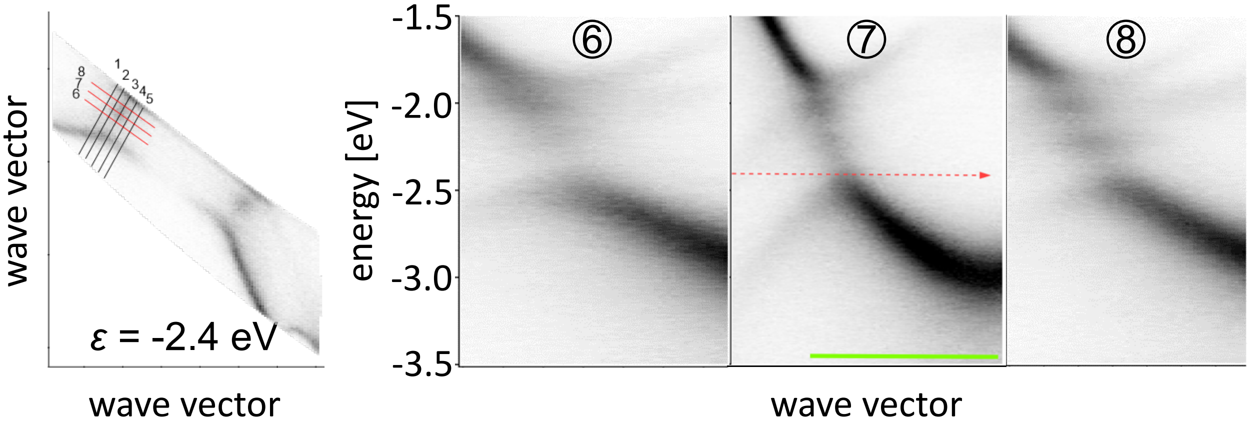

Figure S4: Additional cuts through the secondary Dirac point in the miniband spectrum of large-twist tBLG. The left-most panel presents k-space directions of cuts 1-5, shown in the main text, and cuts 6-8 shown in the panels to the right as marked on top of each of them. The green scale bar corresponds to 0.5 Å^{-1}.

cuts 1-5, this shows that the bands are the closest to each other at the locations indicated with black arrows in Fig. 1(a)(ii). Altogether, cuts 1-8 suggest that constant-energy cuts both above and below ϵ_{sDP} form circular band features with their size shrinking towards the secondary Dirac points. The photointensity is modulated along these circular contours [see Fig. 1(a)(iii)], similarly as it is for the original Dirac points.

Comparison of spectra taken with photons of different energy

In Fig. S5, we compare photoemission spectra along the direction $\boldsymbol{K}_1$ to $\boldsymbol{K}_1^{'}$ for tBLG-C taken with photons with energies 74 eV and 27 eV [the panel for 74 eV photons is the panel shown in the main text in Fig. 3(b)]. While for the lower energy photons we imaged a shorter distance in the reciprocal space, it is clear that the minigap features close to $\boldsymbol{K}_1$ are present in both spectra. Also, no new features appear at 27 eV and similar behaviour of photointensity for both energies contributes to us excluding final state effects, rather than minigaps, as a feasible explanation for our observations.

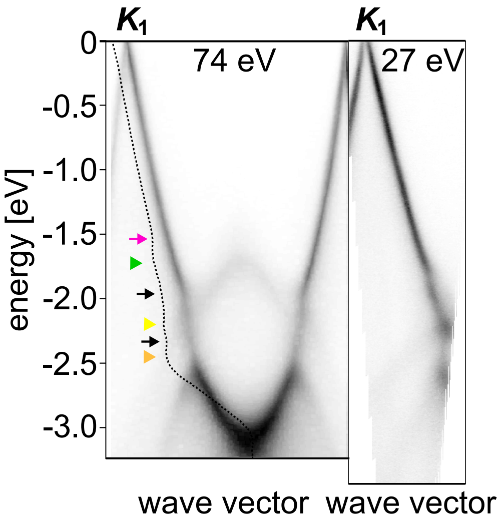

Figure S5: Comparison of k-space cuts along the direction $\boldsymbol{K}_1$ to $\boldsymbol{K}_1^{'}$ taken with photons with energy 74 eV and 27 eV.

Theoretical model of minibands and photoemission spectra

Electronic spectrum of twisted bilayer graphene

We describe twisted bilayer graphene using the Hamiltonian,[6]

$$\boldsymbol{H} = \begin{pmatrix} \boldsymbol{H}_0\left(\frac{\theta}{2}\right) & \boldsymbol{T}(\theta) \\ \boldsymbol{T}^{\dagger}(\theta) & \boldsymbol{H}_0\left(-\frac{\theta}{2}\right) \end{pmatrix}, \tag{1}$$

$$\boldsymbol{H}_0(\theta) = \begin{pmatrix} 0 & -\gamma_0 f(\boldsymbol{R}_\theta \boldsymbol{k}) \\ -\gamma_0 f^*(\boldsymbol{R}_\theta \boldsymbol{k}) & 0 \end{pmatrix},$$

$$f(\boldsymbol{k}) = \exp\left(\frac{i k_y a}{\sqrt{3}}\right) + 2\exp\left(-\frac{i k_y a}{2\sqrt{3}}\right)\cos\left(\frac{k_x a}{2}\right),$$

written in the basis of the sublattice states constructed of carbon p_z orbitals $\phi(\boldsymbol{r}, z) \equiv \phi(x, y, z)$,

$$|\boldsymbol{k}, X\rangle_l = \frac{1}{\sqrt{N}} \sum_{\boldsymbol{R}_l} e^{i\boldsymbol{k}\cdot(\boldsymbol{R}_l + \boldsymbol{\tau}_{X,l})} \phi(\boldsymbol{r} - \boldsymbol{R}_l - \boldsymbol{\tau}_{X,l}, z - z_l), \tag{2}$$

where $\boldsymbol{R}_\theta$ is an operator of clockwise rotation, a is the graphene lattice constant, $\boldsymbol{k} = (k_x, k_y)$ is electron wave vector, $X = A, B$ is the sublattice, $\boldsymbol{R}_l$ are the lattice vectors of layer l, $\boldsymbol{\tau}_{X,l}$ points to the site X in layer l within the unit cell selected by $\boldsymbol{R}_l$ and z_l defines the position of layer l along the z-axis. The diagonal blocks, $\boldsymbol{H}_0$, describe electrons in each of the graphene layers based on the standard tight-binding approach.[8] We write the interlayer coupling following earlier work,[6,7]

$${}_1\langle \boldsymbol{k}', m, |\boldsymbol{T}|\boldsymbol{k}, j\rangle_2 = \sum_{\boldsymbol{G},\boldsymbol{G}'} \tilde{t}(\boldsymbol{k} + \boldsymbol{G}, c_0) e^{-i\boldsymbol{G}\cdot\boldsymbol{\tau}_{j,2}} e^{i\boldsymbol{G}'\cdot\boldsymbol{R}_\theta \boldsymbol{\tau}_{m,1}} \delta_{\boldsymbol{k}+\boldsymbol{G},\boldsymbol{k}'+\boldsymbol{G}'}. \tag{3}$$

The matrix element above can be interpreted in the following way: (1) the Kronecker delta term, $\delta_{\boldsymbol{k}+\boldsymbol{G},\boldsymbol{k}'+\boldsymbol{G}'}$, expresses conservation of crystal momentum and determines the momenta on the top and bottom layers which are coupled (momenta $\boldsymbol{k}$ and $\boldsymbol{k}'$ offset by a moiré reciprocal lattice vector $\boldsymbol{g} = \boldsymbol{G}' - \boldsymbol{G}$); (2) the phase $e^{i(\boldsymbol{G}'\cdot\boldsymbol{R}_\theta\boldsymbol{\tau}_{m,1} - \boldsymbol{G}\cdot\boldsymbol{\tau}_{l,2})}$ is a phase factor associated with the coupling of orbitals m and l as a result of translations by reciprocal lattice vectors in each layer. Qualitatively, these phases describe a continuous transition between regions of AA, AB and BA-like stacking present in the moiré pattern. Finally, the strength of the coupling, $\tilde{t}(\boldsymbol{k}+\boldsymbol{G}, c_0)$, is prescribed by the (total) momentum of the electron tunnelling between the layers. Here, we use parametrization of $\tilde{t}(\boldsymbol{k}+\boldsymbol{G}, c_0)$ as described in Ref. [7] but with $V^0_{pp\pi} = 2.9$ eV to match closer the slope of the Dirac cones. By writing all four matrix elements in the form of a 2×2 matrix, we obtain the interlayer coupling block at the twisted interface, $\boldsymbol{T}(\theta)$,

$$\boldsymbol{T}(\theta) = \sum_{\boldsymbol{G},\boldsymbol{G}'} \tilde{t}(\boldsymbol{k}+\boldsymbol{G}, c_0) \times \begin{pmatrix} e^{i\boldsymbol{G}\cdot\boldsymbol{\tau}} & e^{i(\boldsymbol{G}+\boldsymbol{R}_\theta\boldsymbol{G}')\cdot\boldsymbol{\tau}} \\ 1 & e^{i\boldsymbol{R}_\theta\boldsymbol{G}'\cdot\boldsymbol{\tau}} \end{pmatrix} \delta_{\boldsymbol{k}+\boldsymbol{G},\boldsymbol{k}'+\boldsymbol{G}'}, \tag{4}$$

where $\boldsymbol{\tau} = -(0, a/\sqrt{3})$.

In order to obtain the energy dispersion for a given $\boldsymbol{k}$, we include into our Hamiltonian states coupled to $\boldsymbol{k}$ by the moiré reciprocal vectors which are less than a distance $\frac{28\pi}{3\sqrt{3}r_{AB}} \sin\frac{\theta}{2}$ away from it, compute the matrix elements of $\boldsymbol{H}$ in this truncated basis and diagonalize the resulting matrix numerically. This approach allows to study folding of the graphene dispersions into the moiré Brillouin zone and can be used for any twist angle. However, some caution is required when studying large twists: for given θ, lack of a matching commensurate structure means that the eigenvalues of $\boldsymbol{H}$ cannot be immediately interpreted as representing the electronic band structure as they might not be periodic with respect to the moiré reciprocal vectors. This is not a problem for ARPES simulations because these focus on the momentum-resolved spectral function which is negligible for states folded from distant parts of reciprocal space.

Note that for $\theta = 30°$, subtraction of reciprocal vectors of graphene layers from each other generates two sets of moiré reciprocal vectors and two effective moiré Brillouin zones of the same size but rotated by 30 degrees. Our theoretical model produces the same miniband and ARPES spectra irrespectively of which configuration is used. However, we do not reproduce the experimental results observed for tBLG-C if we explicitly assume twelve-fold rotational symmetry for $\theta = 30°$.

Estimate of moiré scattering gap size

To estimate the magnitudes of the hybridization and moiré-induced gaps observed in ARPES spectra, we use a simplified model capturing coupling between the relevant states on two cones of one layer (shown in blue in Fig. S6) and one cone of the second layer (red in Fig. S6),

$$\tilde{\boldsymbol{H}} = \begin{pmatrix} \epsilon^{(1)}(\boldsymbol{k}) & t & 0 \\ t^* & \epsilon^{(2)}(\boldsymbol{k}) & \tilde{t}^* \\ 0 & \tilde{t} & \epsilon^{(1)}(\boldsymbol{k}-\boldsymbol{G}) \end{pmatrix}. \tag{5}$$

Above, $\epsilon^{(j)}(\boldsymbol{k})$ is the energy of the state in layer j at wave vector $\boldsymbol{k}$, $\boldsymbol{G}$ is a moiré reciprocal vector and t and $\tilde{t}$ express couplings between states in different layers (only such coupling is allowed by the interlayer coupling) which we here treat in full generality as a complex number.

We first discuss the standard case of twist angles below our critical angle, $\theta \ll \theta_c \approx 21.8°$, shown in Fig. S6(a). In this case, the relevant crossing closest to the Dirac points is the one between the blue and red cones. At the wave vector $\boldsymbol{k}$ corresponding to the turning point of this crossing, the energies $\epsilon^{(1)}(\boldsymbol{k})$ and $\epsilon^{(2)}(\boldsymbol{k})$ are equal, $\epsilon^{(1)}(\boldsymbol{k}) = \epsilon^{(2)}(\boldsymbol{k}) = \epsilon_0$. At the same time, $\epsilon^{(1)}(\boldsymbol{k}-\boldsymbol{G}) = \tilde{\epsilon} \ll \epsilon_0$ (cyan surface reaches wave vector $\boldsymbol{k}$ well below the energies shown in the figures; note that attempt to couple to the blue cone translated by a longer superlattice reciprocal vector, for example $2\boldsymbol{G}$, corresponds to a second-order process and

results in a much smaller coupling parameter). We obtain an effective Hamiltonian

$$\tilde{\boldsymbol{H}}_{\mathrm{I}} = \begin{pmatrix} \epsilon_0 & t & 0 \\ t^* & \epsilon_0 & \tilde{t}^* \\ 0 & \tilde{t} & \tilde{\epsilon} \end{pmatrix}. \tag{6}$$

Because $|\tilde{\epsilon}| \gg |\epsilon_0|$, we can project our Hamiltonian onto the two relevant states in the vicinity of the crossing which produces an effective Hamiltonian

$$\boldsymbol{H}_{\mathrm{I}}^{\mathrm{eff}} = \begin{pmatrix} \epsilon_0 & t \\ t^* & \epsilon_0 \end{pmatrix} + \begin{pmatrix} 0 \\ \tilde{t}^* \end{pmatrix} \frac{1}{\epsilon_0 - \tilde{\epsilon}} \begin{pmatrix} 0 & \tilde{t} \end{pmatrix} = \begin{pmatrix} \epsilon_0 & t \\ t^* & \epsilon_0 + \frac{|\tilde{t}|^2}{\epsilon_0 - \tilde{\epsilon}} \end{pmatrix}. \tag{7}$$

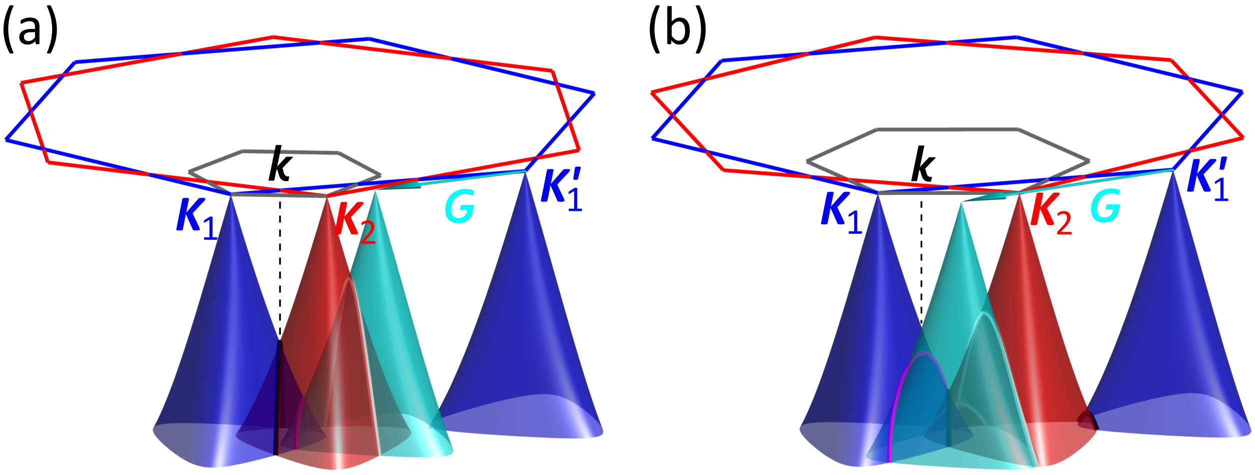

Figure S6: Schematic of crossings between unperturbed dispersions of the top (blue) and bottom (red) graphene layers for (a) 'small' twist angle $\theta = 18°$, (b) large twist of $\theta = 26.5°$. The blue and red hexagons are the BZ of the top and bottom MLG and the corresponding valence band structures in the vicinity of $\boldsymbol{K}_1$, $\boldsymbol{K}_1^{'}$ and $\boldsymbol{K}_2$ are shown with blue and red surfaces. The cyan cone depicts the $\boldsymbol{K}_1^{'}$ states shifted by a moiré reciprocal vector indicated with the cyan arrow (the moiré BZ is shown in gray). Crossings between MLG dispersions are highlighted in black (between two MLG dispersions twisted by θ), magenta (original top MLG dispersion and that translated by a moiré reciprocal vector) and white (bottom MLG and top MLG translated by a moiré reciprocal vector).

This, in turn, produces energy eigenvalues

$$\epsilon = \epsilon_0 + \frac{|\tilde{t}|^2}{2(\epsilon_0 - \tilde{\epsilon})} \pm \left[|t|^2 + \left(\frac{|\tilde{t}|^2}{2(\epsilon_0 - \tilde{\epsilon})} \right)^2 \right]^{\frac{1}{2}}, \tag{8}$$

which for $\frac{|\tilde{t}|^2}{2(\epsilon_0 - \tilde{\epsilon})} \ll |t| \approx 0.11$ eV (as is the case here) simplify to the result obtained neglecting moiré scattering, $\epsilon = \epsilon_0 \pm |t|$ and the hybridization gap $\Delta_{\text{direct}} = 2|t| \approx 0.22$ eV.

In the case of large twist angles, $\theta > \theta_c$, crossing between the $\boldsymbol{K}_1$ cone and $\boldsymbol{K}_1^{'}$ cone translated by the superlattice reciprocal vector $\boldsymbol{G}$ [blue and cyan in Fig. S6(b)] is closer to the Dirac point than the crossing between $\boldsymbol{K}_1$ and $\boldsymbol{K}_2$ [blue and red in Fig. S6(b)]. Using the three-level Hamiltonian, Eq. (5), for the turning point of this crossing, we have $\epsilon^{(1)}(\boldsymbol{k}) = \epsilon^{(1)}(\boldsymbol{k} - \boldsymbol{G}) = \epsilon_0$, $\epsilon^{(2)}(\boldsymbol{k}) = \tilde{\epsilon} \ll \epsilon_0$,

$$\tilde{\boldsymbol{H}}_{\text{II}} = \begin{pmatrix} \epsilon_0 & t & 0 \\ t^* & \tilde{\epsilon} & \tilde{t}^* \\ 0 & \tilde{t} & \epsilon_0 \end{pmatrix}. \tag{9}$$

Notice that in this case, the states that are degenerate at the energy ϵ_0 are not coupled directly as they belong to the same layer. Like before, we can produce an effective Hamiltonian,

$$\boldsymbol{H}_{\text{II}}^{\text{eff}} = \begin{pmatrix} \epsilon_0 & 0 \\ 0 & \epsilon_0 \end{pmatrix} + \begin{pmatrix} t \\ \tilde{t} \end{pmatrix} \frac{1}{\epsilon_0 - \tilde{\epsilon}} \begin{pmatrix} t^* & \tilde{t}^* \end{pmatrix} = \begin{pmatrix} \epsilon_0 + \frac{|t|^2}{\epsilon_0 - \tilde{\epsilon}} & t\tilde{t}^* \\ t^*\tilde{t} & \epsilon_0 + \frac{|\tilde{t}|^2}{\epsilon_0 - \tilde{\epsilon}} \end{pmatrix}, \tag{10}$$

which provides energy eigenvalues

$$\epsilon = \epsilon_0, \qquad \epsilon = \epsilon_0 + \frac{|t|^2 + |\tilde{t}|^2}{\epsilon_0 - \tilde{\epsilon}}. \tag{11}$$

For twisted bilayer graphene, we can assume that $|t| \approx |\tilde{t}| \sim 0.11$ eV.[6] This gives the moiré scattering minigap, $\Delta_{\text{moiré}} = \frac{|t|^2 + |\tilde{t}|^2}{\epsilon_0 - \tilde{\epsilon}} \sim 0.02$ eV (where we took $\epsilon_0 - \tilde{\epsilon} \sim 1$ eV). For

Δ_{direct}, our earlier estimate remains the same to the first order. An improved estimate requires investigation of electronic states coupled by the moiré for a given twist angle to include the most relevant ones in our perturbative description.

Our values for Δ_{direct} and $\Delta_{\text{moiré}}$ compare reasonably well with the gap sizes observed in our ARPES measurements, Fig. 3 of the main text. In Fig. 3(c), the direct hybridization gap, $\Delta_{\text{direct}} \approx 0.25$ eV, is clearly seen at the lowest energies. The Umklapp minigaps visible at higher energies are an order of magnitude smaller, just like our estimates indicate. The slightly larger magnitude of Δ_{direct} and presence of more than one Umklapp minigap is due to the fact that for θ close to 30° several scattering processes, rather than just one as in our three-state model, contribute at energies close to ϵ_0.[9]

ARPES simulations

Using Fermi's golden rule, we write ARPES intensity as[10,11]

$$I \propto \sum_i |M_{f,i}|^2 \, \delta(\omega + \varepsilon_{i,\boldsymbol{k}} - W - \varepsilon_{\boldsymbol{p}_e}), \tag{12}$$

where $M_{f,i}$ is the matrix element describing transition of the electron from the initial state in the crystal in band i to the final state f, ω is the energy of the incident photon, $\varepsilon_{i,\boldsymbol{k}}$ is the energy of an electron in the crystal in band i and with wave vector $\boldsymbol{k}$, $\varepsilon_{\boldsymbol{p}_e}$ is the energy of the photoelectron with momentum $\boldsymbol{p}_e$ and W is the work function of graphene. Within the dipole approximation,

$$M_{f,i} \propto \langle \text{final} | \, \boldsymbol{A} \cdot \boldsymbol{p} \, | \boldsymbol{k}, i \rangle \,, \tag{13}$$

where $\boldsymbol{A}$ is the vector potential of the incident photon, $\boldsymbol{p}$ is the momentum operator, $|\text{final}\rangle$ stands for the final state of the photoelectron and $|\boldsymbol{k}, i\rangle$ denotes the wave function of the electron in the crystal. The latter is a linear combination of the sublattice Bloch states, Eq. (2), corresponding to wave vectors connected by a superlattice reciprocal vector $\boldsymbol{g} =$

$\boldsymbol{G}' - \boldsymbol{G}$,

$$|\boldsymbol{k}, i\rangle = \sum_{\boldsymbol{g}} \sum_{l,X} c^{\boldsymbol{g},i}_{X,l}(\boldsymbol{k})\, |\boldsymbol{R}_{\theta_l}(\boldsymbol{k}+\boldsymbol{g}), X\rangle_l\,, \tag{14}$$

with the coefficients $c^{\boldsymbol{g},i}_{X,l}(\boldsymbol{k})$ provided by diagonalization of the Hamiltonian $\boldsymbol{H}$, Eq. (1). Here, $\boldsymbol{g}$ is the moiré reciprocal superlattice vector. We approximate the final state with a plane wave (justified for incident photon energies above 50 eV[12]) with momentum $\boldsymbol{p}_e = (\boldsymbol{p}_e^{\parallel}, p_e^{\perp})$, so that

$$M_{f,i} \propto \sum_{\boldsymbol{g}} \sum_{l,X} c^{\boldsymbol{g},i}_{X,l}(\boldsymbol{k})\, \langle e^{\frac{i}{\hbar}\boldsymbol{p}_e^{\parallel}\cdot\boldsymbol{r}} e^{\frac{i}{\hbar}p_e^{\perp} z}|\, \boldsymbol{A}\cdot\boldsymbol{p}\, |\boldsymbol{R}_{\theta_l}(\boldsymbol{k}+\boldsymbol{g}), X\rangle_l\,.$$

As long as the photon energy is constant and we are only interested in imaging states with similar magnitude of momentum, $|\boldsymbol{p}| \approx |\boldsymbol{K}|$, the effect of the light-matter interaction, $\boldsymbol{A}\cdot\boldsymbol{p}$, can be captured by a phase factor $e^{i\varphi_{X,l}}$,[13–15]

$$\begin{aligned} M_{f,i} &\propto \sum_{\boldsymbol{g}} \sum_{l,X} e^{i\varphi_{X,l}} c^{\boldsymbol{g},i}_{X,l}(\boldsymbol{k})\, \langle e^{\frac{i}{\hbar}\boldsymbol{p}_e^{\parallel}\cdot\boldsymbol{r}} e^{\frac{i}{\hbar}p_e^{\perp} z}|\boldsymbol{R}_{\theta_l}(\boldsymbol{k}+\boldsymbol{g}), X\rangle_l \\ &= \sum_{\boldsymbol{g}} \sum_{l,X,\boldsymbol{G}_l} e^{i\varphi_{X,l}} c^{\boldsymbol{g},i}_{X,l}(\boldsymbol{R}_{-\theta_l}(\boldsymbol{p}_e^{\parallel}/\hbar + \boldsymbol{G}_l) - \boldsymbol{g}) e^{i\boldsymbol{G}_l\cdot\boldsymbol{\tau}_{X,l}} e^{-\frac{i}{\hbar}p_e^{\perp} z_l} \tilde{\phi}\left(\boldsymbol{R}_{\theta_l}(\boldsymbol{k}+\boldsymbol{g}) - \boldsymbol{G}_l, p_e^{\perp}/\hbar\right), \end{aligned}$$

where

$$\tilde{\phi}\left(\boldsymbol{p}_e^{\parallel}/\hbar, p_e^{\perp}/\hbar\right) = \int d\boldsymbol{r}\, dz\, e^{-\frac{i}{\hbar}\boldsymbol{p}_e^{\parallel}\cdot\boldsymbol{r}} e^{-\frac{i}{\hbar}p_e^{\perp} z} \phi(\boldsymbol{r}, z),$$

is the Fourier transform of the p_z orbital $\phi(\boldsymbol{r}, z)$. Due to the rotational symmetry of the p_z orbital, $\tilde{\phi}\left(\boldsymbol{p}_e^{\parallel}/\hbar, p_e^{\perp}/\hbar\right) = \tilde{\phi}\left(|\boldsymbol{p}_e^{\parallel}/\hbar|, p_e^{\perp}/\hbar\right)$. Moreover, for the given photon energy, ω, and work function, W, we have $p_e^{\perp} \gg |\boldsymbol{p}_e^{\parallel}|$ so that $\tilde{\phi}\left(\boldsymbol{R}_{\theta_l}(\boldsymbol{k}+\boldsymbol{g}) - \boldsymbol{G}_l, p_e^{\perp}/\hbar\right)$ can be approximated by a constant and dropped. Finally, in this work we only study points for which $\boldsymbol{G}_l = 0$. As

a result,

$$I \propto \sum_i \left| \sum_{\boldsymbol{g}} \sum_{l,X} c_{X,l}^{\boldsymbol{g},i}(\boldsymbol{R}_{-\theta_l}\boldsymbol{p}_e^{\parallel} - \boldsymbol{g}) e^{i\varphi_{X,l}} e^{-\frac{i}{\hbar}p_e^{\perp} l c_0} \right|^2 \delta(\omega + \varepsilon_{i,\boldsymbol{k}} - W - \varepsilon_{\boldsymbol{p}_e}), \tag{15}$$

where we used the fact that $z_l = lc_0$. We combine both phases $\exp(i\varphi_{X,l})$ and $\exp(-\frac{i}{\hbar}p_e^{\perp}lc_0)$ into a single factor,

$$e^{i\varphi_{X,l}} e^{-\frac{i}{\hbar}p_e^{\perp} l c_0} = e^{i\alpha_{X,l}}, \tag{16}$$

which we fit to experiment (the experimental data suggests that the phase difference between two neighbouring graphene layers is approximately $e^{i\pi}$).

Finally, we model the Dirac delta in Eq. (15) with a Lorentzian

$$\delta(\omega + \varepsilon_{i,\boldsymbol{k}} - W - \varepsilon_{\boldsymbol{p}_e}) \to \frac{1}{\pi} \frac{\gamma}{(\omega + \varepsilon_{i,\boldsymbol{k}} - W - \varepsilon_{\boldsymbol{p}_e})^2 + \gamma^2}, \tag{17}$$

with half-width-half-maximum γ.

Using our knowledge of the electronic wave functions, we can also compute the single particle spectral weight of the wave function of band i at wave vector $\boldsymbol{k}$,

$$S_i(\boldsymbol{k}, \epsilon_{\boldsymbol{k},i}) = \sum_l |{}_l\langle \boldsymbol{k} | \boldsymbol{k}, i\rangle|^2, \tag{18}$$

where $|\boldsymbol{k}\rangle_l$ is the unperturbed eigenstate of layer l at wave vector $\boldsymbol{k}$. In the absence of interlayer coupling, the wave function $|\boldsymbol{k}, i\rangle$ is identical to one of the unperturbed states $|\boldsymbol{k}\rangle_l$ and $S_i(\boldsymbol{k}, \epsilon_{\boldsymbol{k},i}) = 1$. In the presence of the coupling, $S_i(\boldsymbol{k}, \epsilon_{\boldsymbol{k},i})$ provides us information about the proportion of the original unperturbed states at $\boldsymbol{k}$ contributing to the moiré superlattice wave function $|\boldsymbol{k}, i\rangle$. In Fig. S7, we compare the experimental and theoretical photoemission spectra from Fig. 3(c) of the main text with $S_i(\boldsymbol{k}, \epsilon_{\boldsymbol{k},i})$. Notice that all the minigaps we discuss in the main text are already present in $S_i(\boldsymbol{k}, \epsilon_{\boldsymbol{k},i})$ and hence are true minigaps and

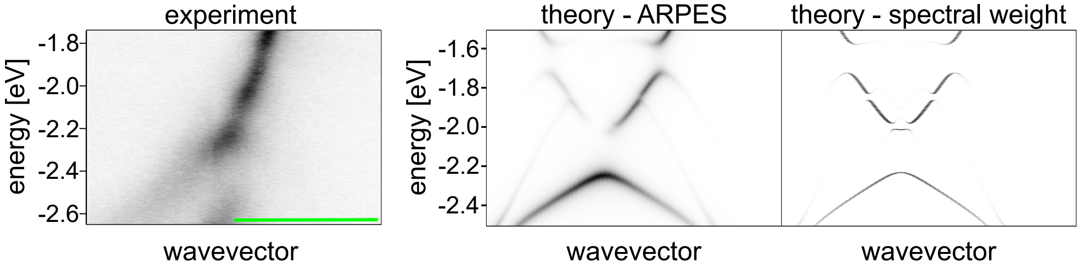

Figure S7: Comparison of the experimental and theoretical photoemission spectra from Fig. 3(c) of the main text with $S(\boldsymbol{k}, \epsilon_{\boldsymbol{k},i})$ for the same range of energies and wave vectors as the theoretical ARPES simulation.

not photointensity modulation features arising due to Berry phase or final state effects (here, we use $S_i(\boldsymbol{k}, \epsilon_{\boldsymbol{k},i})$ to study the minibands because plotting all $\epsilon_{\boldsymbol{k},i}$ directly for a general twist angle includes all of the folded and moiré-coupled bands, most of which are weakly coupled and yet complicate interpretation of the spectrum).

Matching of simulations to experimental data

In Fig. 3(c) of the main text, the energy windows of the simulation and experimental data are not exactly the same. Similarly, in Fig. 1(a) of the main text, the values of the energies as given correspond to the energies of the experimental cuts while the simulated maps have been selected to display similar features and correspond to an energy within $\sim$ 200 meV away from the experimental value. There are several reasons for the mismatch between the simulation and experiment: (i) within our theoretical model, the individual graphene layers are described using a single parameter, the nearest neighbour hopping, γ_0, which makes matching experimental features across the energy range of over 3 eV away from the Dirac points difficult;[16] (ii) the experimental energy resolution is about 75-80 meV [except the closeup in Fig. 2(c) for which the resolution is $\sim$ 45 meV]; (iii) the accuracy with which we can determine the positions of the Dirac points of both layers (which specify experimental zero of energy) is $\sim$ 40 meV; (iv) finally, nonuniformity of the detector dispersion introduces nonlinear transformation of the position of the feature as measured by the detector to binding energy which leads to an additional shift of up to $\sim$ 100 meV at energies $\sim$ 2 eV below the

Dirac point as compared to energies close to the Dirac point; this shift depends on the details of a particular measurement (for example, photon energy). Given these uncertainties, we are satisfied with the matching of the features between theory and experiment – attempts to fine-tune the theoretical model to produce a closer quantitative correspondence would add little to the understanding of the observed phenomena.

References

(1) Wang, D.; Chen, G.; Li, C.; Cheng, M.; Yang, W.; Wu, S.; Xie, G.; Zhang, J.; Zhao, J.; Lu, X.; Chen, P.; Wang, G.; Meng, J.; Tang, J.; Yang, R.; He, C.; Liu, D.; Shi, D.; Watanabe, K.; Taniguchi, T.; Feng, J.; Zhang, Y.; Zhang G. Thermally Induced Graphene Rotation on Hexagonal Boron Nitride. *Phys. Rev. Lett.* **2016**, *116*, 126101.

(2) Mentes, T. O.; Zamborlini, G.; Sala, A.; Locatelli, A. Cathode Lens Spectromicroscopy: Methodology and Applications. *Beilstein J. Nanotechnol.* **2014**, *5*, 1873-1886.

(3) Bauer, E. *Surface Microscopy with Low Energy Electrons*. Springer: New York, 2014, 409-464.

(4) Dudin, P.; Lacovig, P.; Fava, C.; Nicolini, E.; Bianco, A.; Cautero, G.; Barinov, A. V. Angle-Resolved Photoemission Spectroscopy and Imaging with a Submicrometre Probe at the SPECTROMICROSCOPY-3.2L Beamline of Elettra. *J. Synchrotron Radiat.* **2010**, *17*, 445-450.

(5) Ahn, S. J.; Moon, P.; Kim, T.-H.; Kim, H.-W.; Shin, H.-C.; Kim, E. H.; Cha, H. W.; Kahng, S.-J.; Kim, P.; Koshino, M.; Son, Y.-W.; Yang, C.-W.; Ahn, J. R. Dirac Electrons in a Dodecagonal Graphene Quasicrystal. *Science* **2018**, *361*, 782-786.

(6) Bistritzer, R.; MacDonald, A. H. Moiré Bands in Twisted Double-Layer Graphene. *Proc. Natl. Acad. Sci. U.S.A* **2011**, *108*, 12233-12237.

(7) Koshino, M. Interlayer Interaction in General Incommensurate Atomic Layers. *N. J. Phys.* **2015**, *17*, 015014.

(8) Castro Neto, A. H.; Guinea, F.; Peres, N. M. R.; Novoselov, K. S.; Geim, A. K. The Electronic Properties of Graphene. *Reviews of Modern Physics* **2009**, *81*, 109-162.

(9) Moon, P.; Koshino, M.; Son, Y.-W. Quasicrystalline Electronic States in 30° Rotated Twisted Bilayer Graphene. *Phys. Rev. B* **2019**, *99*, 165430.

(10) Mucha-Kruczynski, M.; Tsyplyatyev, O.; Grishin, A.; McCann, E.; Fal'ko, V. I.; Bostwick, A.; Rotenberg, E. Characterization of Graphene through Anisotropy of Constant-Energy Maps in Angle-Resolved Photoemission. *Phys. Rev. B* **2008**, *77*, 195403.

(11) Mucha-Kruczynski, M.; Wallbank, J. R.; Fal'ko, V. I. Moiré Miniband Features in the Angle-Resolved Photoemission Spectra of Graphene/hBN Heterostructures. *Phys. Rev. B* **2016**, *93*, 085409.

(12) Gierz, I.; Lindroos, M.; Hochst, H.; Ast, C. R.; Kern, K. Graphene Sublattice Symmetry and Isospin Determined by Circular Dichroism in Angle-Resolved Photoemission Spectroscopy. *Nano Letters* **2012**, *12*, 3900-3904.

(13) Liu, Y.; Bian, G.; Miller, T.; Chiang, T.-C. Visualizing Electronic Chirality and Berry Phases in Graphene Systems Using Photoemission with Circularly Polarized Light. *Phys. Rev. Lett.* **2011**, *107*, 166803.

(14) Gierz, I.; Henk, J.; Hochst, H.; Ast, C. R.; Kern, K. Illuminating the Dark Corridor in Graphene: Polarization Dependence of Angle-Resolved Photoemission Spectroscopy on Graphene. *Phys. Rev. B* **2011**, *83*, 121408.

(15) Hwang, C.; Park, C.-H.; Siegel, D. A.; Fedorov, A. V.; Louie, S. G.; Lanzara, A. Direct Measurement of Quantum Phases in Graphene *via* Photoemission Spectroscopy. *Phys. Rev. B* **2011**, *84*, 125422.

(16) Jung, J.; MacDonald, A. H. Tight-Binding Model for Graphene π-Bands from Maximally Localized Wannier Functions. *Phys. Rev. B* **2013**, *87*, 195450.
